# Surgical approach could be a major factor in placenta previa outcome: A comparative retrospective study

**DOI:** 10.1097/MD.0000000000036437

**Published:** 2023-11-24

**Authors:** Ala N. Uwais, Anas O. Satari, Marleen M. Hijazin, Ahmed A. Al-abadleh, Sahel W. Haddadin

**Affiliations:** a Department of Obstetrics and Gynecology, Faculty of Medicine, Mutah University, AL-Karak, Jordan; b Faculty of Medicine, Mutah University, AL-Karak, Jordan; c Al-Karak Governmental Hospital, AL-Karak, Jordan; d Department of General Surgery, King Hussien Medical Hospital, Royal Medical Services, Amman, Jordan.

**Keywords:** caesarean hysterectomy, caesarean section, hysterectomy, placenta previa

## Abstract

Placenta previa is associated with high morbidity and mortality rates due to major hemorrhage during surgery. Thus, a standard surgical approach with a low risk of morbidity is required. This study aimed to propose surgical steps for placenta previa with scarred uterus. All deliveries at the Al-Karak governmental hospital between January 2019 and January 2022 were retrospectively reviewed. Placenta previa cases were divided into 2 groups according to management. Group A was managed by incising the uterus at the level of the fundus to avoid disrupting the placenta, whereas group B was managed by opening the lower uterine segment and delivering the baby through the placenta after the incision. A total of 26 cases with placenta previa were included in this study. Group A (n = 12) was managed by avoiding the placenta and group B (n = 14) was managed by opening through the placenta. No differences were noted between the 2 groups regarding demographics. Patients who underwent the suggested surgical approach (Group A) had less blood loss (median = 775 cc), whereas Group B (median = 1700 cc) (*P* = .001) had significantly higher blood loss. The duration of hospital stay was significantly shorter in Group A (median = 2 days) than in Group B (median = 6 days) (*P* = .000). Incising the upper uterine segment to avoid the placenta may lead to better outcomes in terms of blood loss and its consequences.

## 1. Introduction

Placenta previa is a major cause of bleeding during the third trimester of pregnancy.^[1]^ Its incidence has increased over the past few years owing to an increase in the cesarean section (CS) rate. Adherent placenta (placenta accreta) occurs in one of 533 pregnancies.^[2]^ Placenta accreta is currently the most frequent indication for cesarean hysterectomy and is associated with major hemorrhage, with almost 90% of women requiring blood transfusion and 40% requiring more than 10 units of blood.^[3–5]^ The maternal bladder is often involved in placental invasion, which increases morbidity and mortality.^[6]^ Various surgical techniques have been described to perform cesarean hysterectomy safely and expeditiously, reduce hemorrhage, and improve outcome, including a posterior retrograde hysterectomy approach via the pouch of Douglas, or inserting an intravascular balloon in the anterior division of the internal iliac artery.^[7–9]^

The rate of cesarean hysterectomy for women with placenta previa late in the third trimester is high and inversely correlates with the distance from the placental edge to the internal cervical os on transvaginal sonography, also with women with a history of at least 1 cesarean section, or if the lower placental edge is thing and the lacuna is shown in the placenta.^[10–12]^ However, cesarean delivery for placenta previa is a major risk factor for severe postpartum hemorrhage^[13]^; thus, it should be approached assuming the worst surgical scenario. The exact incidence of maternal mortality related to placenta previa and its complications is unknown, but it has been reported to be as high as 6% to 7% in case series and surveys.^[14,15]^

This is a comparative retrospective study of a specific surgical approach for placenta previa, which aims to compare the proposed method with the conventional cesarean method.

## 2. Methods

### 2.1. Patients and data collection

Retrospectively, extraction of data was carried out from patients records for 40 females who were diagnosed from January 2019 to January 2022 in Al-Karak governmental hospital with placenta previa (diagnosis was based on ultrasonography). Any case of posterior placenta previa or placenta previa with an unscarred uterus were excluded. Patient contact information was collected, and consent was obtained via phone calls. Although the authors were unfortunate, obtaining informed consent from patients was not possible, since the majority of participants had difficulties reaching the hospital. Despite this, this study was approved by the Institutional Review and Ethics Committee of the Faculty of Medicine, Mutah (Reference No. 9013070 date: October 15, 2021). The diagnosis of placenta previa was established based on the last transvaginal or transabdominal ultrasonographic examination. Data regarding age, parity, number of previous CS, duration of hospitalization, duration of intensive care unit (ICU) stay, blood loss during delivery, number of packed red blood cells (PRBC) given, and hysterectomy status were collected from hospital records for each patient. Subjects were divided into 2 groups according to the surgical approach by which delivery was performed: the first (Group A) was managed by the same surgeon, and the second (Group B) was managed by other surgeons in the hospital.

### 2.2. Surgical approach

In Group A, the operation started with a midline incision extending above the umbilicus to open the uterus at the fundus. The bladder was then dissected using Mayo scissors by opening the broad ligament laterally to reach the vesicouterine space to introduce the index finger between the bladder anteriorly and the cervix posteriorly. Subsequently, the adhesion bands between the dome of the bladder and the uterus were cut. Following that, the bladder was dissected until the glossy white appearance of the cervix was observed. Occasionally, bleeding occurs during bladder dissection; however, it is controlled by compression. Next, the uterus was opened at the level of the fundus and the baby was delivered. Following delivery, manual removal of the placenta was attempted; however, hysterectomy was performed if the placenta was adherent. In group B, a conventional cesarean delivery was performed. For the uterine repair, double and triple layers were used.

### 2.3. Data analysis

Categorical variables were expressed as frequencies and percentages, and continuous variables were expressed as medians (25^th^–75^th^ percentiles) due to the small sample size. The Mann–Whitney *U* test was used to compare medians of continuous variables, and chi–square of test was used to explore differences in categorical variables. Significant *P* value was set at < .05, and SPSS version 25.0 was used to analyze data.

## 3. Results

### 3.1. General features

**Figure 1.**
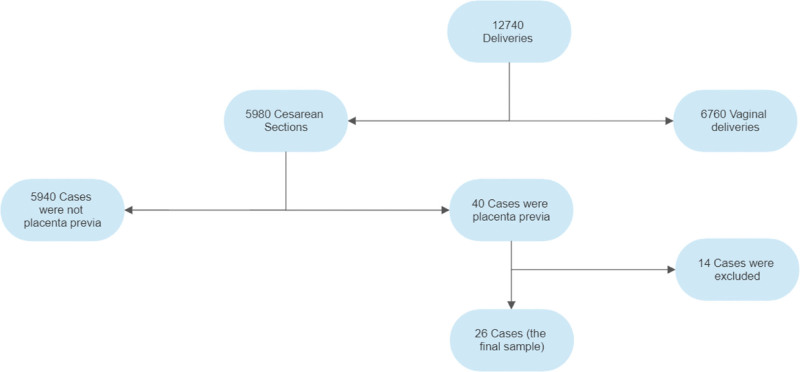
A flow chart demonstrating the sample included in the study.

In the mentioned period in Al-Karak governmental hospital, a total of 12,740 females were delivered; of these, 5980 were cesarean deliveries, of which 40 were placenta previa cases. After excluding 9 cases of posterior placenta previa and 5 cases of placenta previa with an unscarred uterus, only 26 patients were enrolled in this study (Fig. 1).

Table 1 demonstrates that the median age of the sample was 37.0 (30–40) years, and both the median parity and median value of previous CS were 3.0 (3.0–4.2) and 2.0 (1.0–3.0) respectively. In addition, the median time in days that the patients spent in both the obstetrics and gynecology ward and the ICU unit was 3.5 (2.0–6.0) and 0.0 (0.0–2.0) days respectively. Moreover, the median blood loss of the included cases was 1175.0 (787.5–1712.5) cc, and the median number of PRBC given was 3.5 (0.7–6.0) packs. Of the total cases, 12 (46.2%) were subjected to cesarean hysterectomy, while 14 (53.8%) were not. Finally, 12 (46.2%) cases were managed by surgical approach A and 14 (53.8%) were managed by surgical approach B.

**Table 1:** General characteristic features for the study sample.

	Overall (n = 26)
Age (yr)	37.0 (30–40)
Parity (births)	3.0 (3.0–4.2)
Previous CS (times)	2.0 (1.0–3.0)
Duration of hospitalization (d)	3.5 (2.0–6.0)
ICU stay duration (d)	0.0 (0.0–2.0)
Blood loss (cc)	1175.0 (787.5–1712.5)
PRBCs given (packs)	3.5 (0.7–6.0)
Hysterectomy	
No, n (%)	14 (53.8)
Yes, n (%)	12 (46.2)
Surgical group	
Group A, n (%)	12 (46.2)
Group B, n (%)	14 (53.8)

CS = cesarean section, ICU = intensive care unit, PRBC = packed red blood cells.

### 3.2. Effect of surgical approach on clinical outcome

To explore whether there was a statistically significant median difference between the 2 groups, the Mann–Whitney *U* test was used. The results showed that the median number of admission days in group B (median = 6) was significantly higher than that in group A (median = 2) (U = 10, *P* = .001). As for ICU stay, in group A, only 1 patient required ICU admission (median = 0); however, in group B, 7 patients required an ICU stay (median = 1); this difference was statistically significant (U = 45.50, *P* = .015). Results for blood loss showed that the median blood loss of group B (median = 1700 cc) was significantly higher than that of group A (median = 775 cc) (U = 20.0, *P* = .001). Similarly, the median number of PRBC given to group B (median = 5 units) was significantly higher than that for group A (median = 1 unit) (U = 29.5, *P* = .005). No statistically significant differences were observed between the groups in terms of age, parity, or number of previous CS (*P* > .05) (Table 2).

**Table 2:** Mann–Whitney *U* test for median differences between two groups.

	Surgical group	Median (25^th^–75^th^ percentiles)	U value	*P* value
Age (yr)	Group A	36.5 (30.5–41.0)	74.5	.623
	Group B	37.5 (30.0–39.2)		
Parity (births)	Group A	3.5 (3.0–5.0)	59.0	.173
	Group B	3.0 (2.7–3.2)		
Previous CSs (times)	Group A	3.0 (2.0–3.7)	50.5	.073
	Group B	2.0 (1.0–3.0)		
Duration of hospitalization (d)	Group A	2.0 (2.0–2.7)	10.0	.001
	Group B	6.0 (3.7–8.0)		
ICU stay duration (d)	Group A	0.0 (0.0–0.0)	45.5	.015
	Group B	1.0 (0.0–3.2)		
Blood loss (cc)	Group A	775.0 (700.0–1187.5)	20.0	.001
	Group B	1700.0 (1100–2537.5)		
PRBCs given (packs)	Group A	1.0 (0.0–2.7)	29.5	.005
	Group B	5.0 (3.7–10.5)		

CS = cesarean section, ICU = intensive care unit, PRBC = packed red blood cells.

A chi–square test was conducted to study the association between cesarean hysterectomy and the surgical approach performed. The results showed no statistically significant association between the 2 variables (*P* > .05), suggesting that hysterectomy was not dependent on the surgical approach used (Table 3).

**Table 3:** Chi–square test result of association between hysterectomy and surgical approach.

		Caesarean hysterectomy		X^2^	*P* value
		No	Yes		
Surgical group	Group A	8 (57.1%)	4 (33.3%)	1.4	.225
	Group B	6 (42.9%)	8 (66.7%)		

In our supplementary analyses, we found a weak negative correlation between gestational age and bleeding amount (r = −0.196), no significant relationship between operation time and bleeding (*R* = 0.0043), and no discernible difference in bleeding based on placental position (*P* value = 0.433).

## 4. Discussion

The usual surgical sequence during CS for placenta previa starts with delivery of the baby through the placenta. If the placenta is adherent, the surgeon may decide to perform hysterectomy, at this point blood loss will be significant due to placental incision and the time taken for bladder dissection.^[9,16–18]^

In this study, we compared the outcomes of 2 approaches (transecting or avoiding placental incision) for cesarean delivery in patients with placenta previa. The compared outcomes were the amount of blood loss, packed red RBCs required, ICU admission, and length of hospital stay. The results showed that avoiding placental transection decreased blood loss, blood transfusion requirement, ICU admission, and length of hospital stay.

In this study, primigravida’s were excluded because we did not perform classical CS on an unscarred uterus because the placenta is usually easily separated in such cases. However, there is a risk of major hemorrhage with placental transection. Moreover, patients with posterior placenta previa were excluded, as our approach focuses on avoiding placental incision; therefore, opening the lower segment is feasible in these cases.

Few studies have correlated the amount of blood loss during cesarean delivery through the placenta. One study showed that anterior placenta previa is a significant risk factor for major blood loss during surgery.^[19]^ Moreover, a retrospective study with a larger sample size compared transection and avoiding the placenta during surgery. The study concluded that avoiding the placenta decreased blood loss and the need for blood transfusion.^[20]^

**Figure 2.**
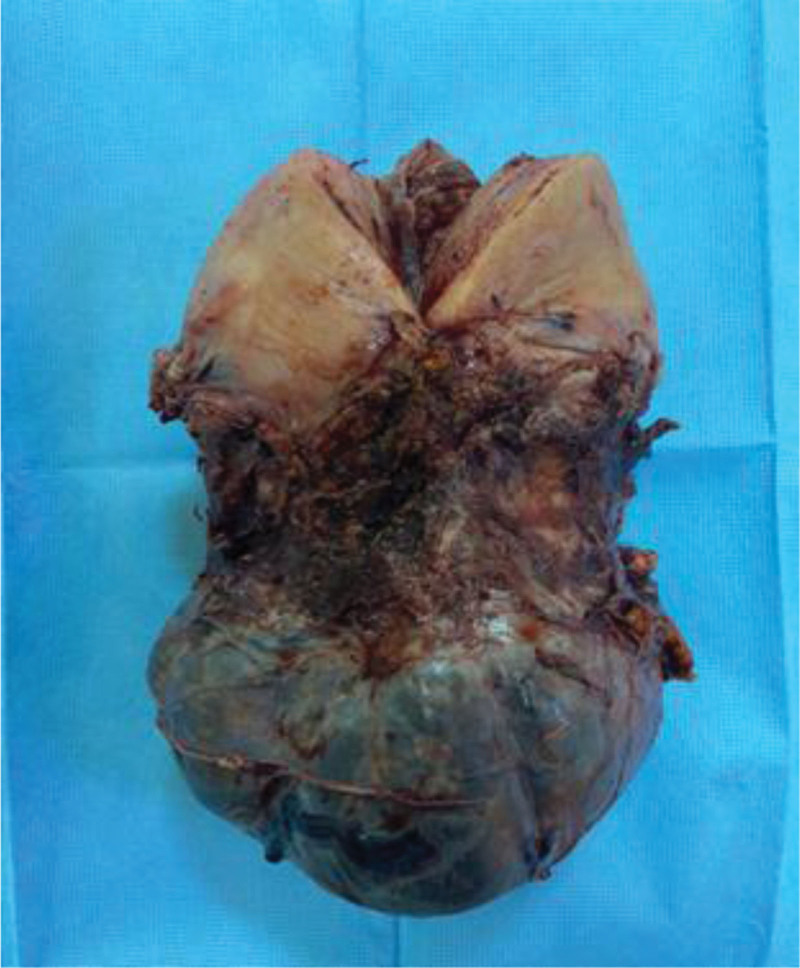
Depicts the classical incision at the fundus.

The surgical approach described in this study focused on 2 concepts. First, dissecting the bladder and free adhesions around the lower uterine segment, assuming that the patient may have placenta accreta, and hysterectomy may be required. Second, the placenta was avoided by making a high vertical incision at the fundus (Fig. 2) to decrease blood loss. The same concept was used by Saha et al^[2]^ in 12 suspected placenta accreta cases, which resulted in less hemorrhage and no reported cases of bladder or ureteric injury.

In reviewing the guidelines for the surgical approach in placenta previa treatment, no committee has provided clear guidance on the surgical technique. The Royal College of Obstetricians and Gynecologists recommends avoiding the placenta and using intraoperative ultrasound to localize it.^[21]^ In this described surgical technique, we were keen to open the abdominal wall by midline incision, as this improves access to the upper segment, allowing the uterine incision to be made away from the placenta. Moreover, the midline incision enables better access to the broad ligament, enabling lateral dissection of the bladder where there are no adhesions.

We recommend opening the upper uterine segment vertically for 2 main reasons. First, the transverse incision may have extended to the broad ligament. Second, the uterine smooth muscles are arranged in a vertical pattern^[22]^; thus, a high vertical incision transects fewer muscle fibers, making it easier to repair. Verspyck et al^[20]^ compared 2 surgical methods, one of which aimed to circumvent the placenta to avoid cutting through it. Avoiding the placenta was possible in 67% of cases.^[1]^ In our 12 cases, avoiding the placenta was possible even if the placenta extended to the anterior wall; however, in some cases, the surgeon may have to open the fundus. Although some may raise concerns about subsequent pregnancies and an increased expected risk of uterine rupture and adhesions, the value of significantly decreasing maternal mortality and morbidity outweighs this. In particular, this approach is not used in cases with an unscarred uterus or posterior placenta previa.

As in any retrospective study, the frequency of each outcome was not calculated in this study. Hence, the results of our study can be applied to well-designed studies with large sample sizes. Moreover, our study has some limitations and potential bias. First, the 2 approaches were performed by different surgeons; thus, surgical experience may have influenced the outcomes rather than the surgical approach itself. Second, at our hospital, there is no clear standardized method to calculate blood loss, which depends solely on the surgeons estimation; however, the number of packed RBCs is suggestive of the amount of blood loss during surgery. Also, due to small sample size, we have not been able to differentiate and analyze the associations with bleeding volumes and the different types of placenta accreta, including increta, acreta, and percreta.

## 5. Conclusion

Restoring anatomy before uterine incision and avoiding placental transection may reduce blood loss.

## Author contributions

**Conceptualization:** Ala N. Uwais.

**Formal analysis:** Anas O. Satari.

**Investigation:** Marleen M. Hijazin.

**Project administration:** Ala N. Uwais.

**Supervision:** Sahel W. Haddadin.

**Writing – original draft:** Ala N. Uwais, Marleen M. Hijazin, Ahmed A. Al-abadleh.

**Writing – review & editing:** Anas O. Satari, Ahmed A. Al-abadleh.
